# alfaNET: A Database of Alfalfa-Bacterial Stem Blight Protein–Protein Interactions Revealing the Molecular Features of the Disease-causing Bacteria

**DOI:** 10.3390/ijms22158342

**Published:** 2021-08-03

**Authors:** Raghav Kataria, Rakesh Kaundal

**Affiliations:** 1 Department of Plants, Soils, and Climate, College of Agriculture and Applied Sciences, Utah State University, Logan, UT 84322, USA; raghav.kataria@usu.edu; 2 Bioinformatics Facility, Center for Integrated BioSystems, College of Agriculture and Applied Sciences, Utah State University, Logan, UT 84322, USA; 3 Department of Computer Science, College of Science, Utah State University, Logan, UT 84322, USA

**Keywords:** alfalfa, *Pseudomonas syringae* ALF3, bacterial stem blight, effectors, protein–protein interactions, interolog, database

## Abstract

Alfalfa has emerged as one of the most important forage crops, owing to its wide adaptation and high biomass production worldwide. In the last decade, the emergence of bacterial stem blight (caused by *Pseudomonas syringae* pv. *syringae* ALF3) in alfalfa has caused around 50% yield losses in the United States. Studies are being conducted to decipher the roles of the key genes and pathways regulating the disease, but due to the sparse knowledge about the infection mechanisms of *Pseudomonas*, the development of resistant cultivars is hampered. The database alfaNET is an attempt to assist researchers by providing comprehensive *Pseudomonas* proteome annotations, as well as a host–pathogen interactome tool, which predicts the interactions between host and pathogen based on orthology. alfaNET is a user-friendly and efficient tool and includes other features such as subcellular localization annotations of pathogen proteins, gene ontology (GO) annotations, network visualization, and effector protein prediction. Users can also browse and search the database using particular keywords or proteins with a specific length. Additionally, the BLAST search tool enables the user to perform a homology sequence search against the alfalfa and *Pseudomonas* proteomes. With the successful implementation of these attributes, alfaNET will be a beneficial resource to the research community engaged in implementing molecular strategies to mitigate the disease. alfaNET is freely available for public use at http://bioinfo.usu.edu/alfanet/.

## 1. Introduction

Alfalfa (*Medicago sativa* L.) is one of the major forage crops in the United States and other countries around the world. The crop is widely cultivated owing to its high biomass production, role in biological nitrogen fixation and soil conservation, and high nutritional value as animal feed [1]. Besides these, various essential secondary metabolites (such as lignin, saponins, and flavonoids) are synthesized in alfalfa. These secondary metabolites have been reported to provide plants resistance against biotic and abiotic stresses [2]. An early accumulation of lignin was observed in the cell wall during the plant defense mechanism against the disease [3]. The intermittent occurrence of bacterial stem blight of alfalfa is common in the central and western US, with many recent reports in Europe, Australia, and western Iran [4]. The disease has been reported to cause a yield loss of 40 to 50% in the first crop harvest [5]. The Gram-negative bacteria, *Pseudomonas syringae*, is classified into at least sixty pathogenic strains, characterized according to their target host. The bacteria possess virulence factors such as a type III secretion system (T3SS) to infect its host and hijack the host transcription machinery [6]. *P. syringae* pv. *syringae* ALF3, the causal organism of bacterial stem blight, infects its host through the frost mechanism because of the presence of a unique ice-nucleation protein (INP) on the outer membrane of the bacteria, which serves as the nuclei for the disease initiation [7]. The disease occurs in two successive phases: localized foliar necrosis (blight) and systemic vascular wilt. In the initial stage, the bacterial penetration forms water-soaked lesions at the frost injury sites, which further extends and produces dried bacterial exudate throughout the stem. The diseased plants are usually dwarfs, with slender stems that blacken upon maturity [8].

Various research groups in the past have elucidated the genetics behind the interaction mechanism of *P. syringae* with its plant hosts in model plants [9], which involves gene-for-gene resistance, thus triggering a cascade of defense-related signals which eventually lead to disease resistance. Such incompatible host–pathogen interactions also initiate a hypersensitive response (HR) in plants [10]. However, information on the disease resistance mechanism in alfalfa is limited. Researchers in the past have isolated bacteria with symptoms of bacterial stem blight from alfalfa and identified strain ALF3 as *P. syringae* pv. *syringae* on the basis of the 16S rDNA sequence and PCR amplification of syrB for toxin production [11]. Following the genome sequencing of the strain ALF3 [8], it was found to be pathogenic in various plants such as *Medicago truncatula*, pear leaves, and bean seed pods [11]. Significant differentially expressed genes and processes associated with the host defense mechanism have also been proposed in alfalfa infected with bacterial stem blight [12]. Studies are being conducted to decipher the role of key genes and pathways regulating the disease, but the sparse availability of knowledge about the infection mechanism of *P. syringae* hampers the development of resistant cultivars.

The protein–protein interactions (PPIs) between a host and pathogen play a crucial role in the infection process and the initiation of a defense response against pathogen attack. Therefore, studying the PPI network between host and pathogen proteins helps us understand the underlying infection mechanism [13]. The identification of potential alfalfa proteins targeted by *P. syringae* will further bolster the management of the disease. There are plentiful resources from which to retrieve host–pathogen PPI data for many plant species [14], but so far no open resources have been developed for the retrieval of information for alfalfa inter-species PPIs. The implementation of host–pathogen interaction databases containing various functional analysis features supports the development of novel disease-resistant cultivars [15,16]. Databases such as Pseudomonas Genome Database [17], which provide information about the genome sequences and annotation of different *Pseudomonas* species, are implemented. LegumeIP [18], a database of model legumes, provides comparative genomics and transcriptomics information of various legume crops. However, no tool for the prediction of host-pathogen PPI exists for alfalfa-*P. syringae* systems.

In the present study, we aim to develop an interactomic resource, alfaNET, to provide the research community with a platform to study *Pseudomonas*–alfalfa inter-species interactions. The database holds information about annotations of *P. syringae*, along with the subcellular localization information of the pathogen proteins. Furthermore, alfaNET implements a host–pathogen interactomics tool, allowing the users to exploit the predicted PPIs between alfalfa and *P. syringae*. The tool promises a user-friendly interface, providing users with an enriched network visualization framework for better understanding the host–pathogen systems. alfaNET is freely available for public use at http://bioinfo.usu.edu/alfanet/.

## 2. Results

### Database Architecture and Implementation

**Figure 1:**
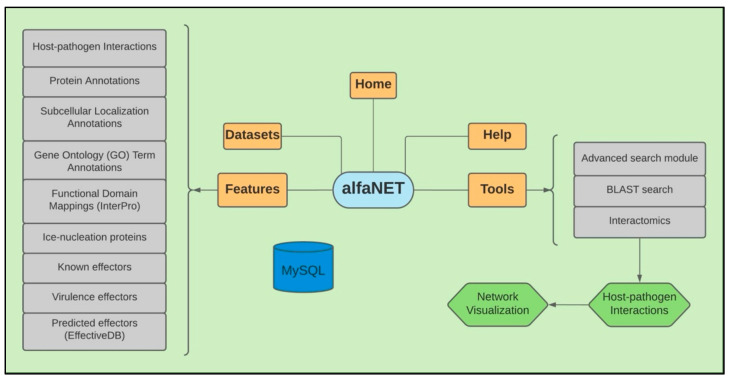
Sitemap architecture of the alfaNET database.

The sitemap architecture of the alfaNET database is depicted in Figure 1. The tool is hosted on a Linux virtual node which is located in a high-performance computing (HPC) cluster. The implementation of server code is performed in PHP and functions through an Apache server. We used Bootstrap and various JavaScript plugins to develop the front-end, thus enhancing the visual appearance. For data retrieval, separate pages are available in the database, whereby the user can query information for the required species. The user can further filter this information using keywords and download it in a tab-delimited format, which can be accessed in spreadsheet editors such as Microsoft Excel. Furthermore, the database provides users with a BLAST utility that implements alfalfa and *P. syringae* pv. *syringae* ALF3 protein sequences as BLAST databases at the backend. We also employed BlasterJS [19] for displaying the BLAST alignment visualization.

**Figure 2:**
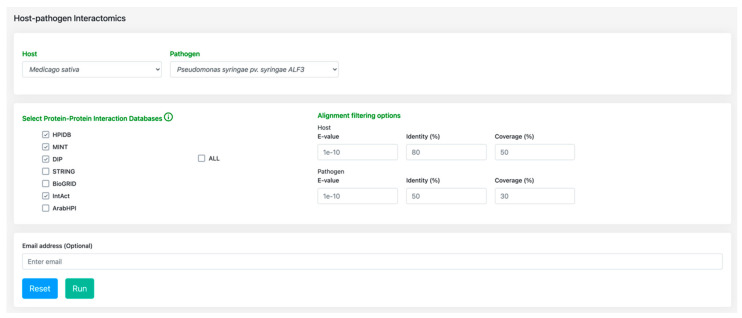
Interface of the host-pathogen interactome tool.

In addition to the utilities mentioned above, the major functionality of alfaNET is the host–pathogen interactome tool (Figure 2), which implements an optimized interolog approach to predict the host-pathogen interactome of the *Medicago* and *Pseudomonas* species using various in-house R scripts and SQL operations in a fraction of a second. The interface of the interactome tool is designed in such a manner that it allows users to select various protein-protein interaction databases to be used, while also providing the alignment filtering options, allowing them to filter the BLAST alignments of both the host and pathogen datasets by adjusting the parameters such as E-value, identity, (%) and coverage (%). On job submission by the user, a unique identifier (Job ID) is assigned, which the user can access to check the job status (queried, active, or completed). After the completion of the job, the results are displayed in an enhanced tabular format with advanced filtering options, enabling users to sort the result data into columns or filter using keywords. The results are available to be downloaded in different formats, such as in pdf or excel, or they can be copied in the clipboard. Additionally, the e-mail address feature in this tool notifies the user of the completion of a job. To visualize the protein interaction networks, SigmaJS [20] is implemented at the backend with various layout options, links, and buttons, thus enabling the user to export the network in JSON format. The network visualization utility also has an option for saving the network in SVG format, which is another advantage, allowing the user to generate high-quality images for publications. All the result data, including HPI predictions, features, and network files, are stored for a period of 30 days on the backend server. Access to the saved results can be further extended upon the user’s request.

## 3. Discussion

### 3.1. Features Search: A Central Resource to Retrieve P. syringae Annotations

alfaNET implements nine search modules to represent the result data from the host–pathogen PPI prediction analysis pipeline. From these, eight modules consist of different annotation categories (*Protein Annotations*, *Subcellular Localization Annotations*, *Gene Ontology (GO) Term Annotations*, *Functional Domain Mappings (InterPro)*, *Ice-nucleation proteins*, *Known effectors*, *Virulence effectors*, *and Predicted effectors from EffectiveDB*) of *P. syringae* proteins. Furthermore, the module ‘*Host-pathogen interactions*’ includes the alfalfa proteins involved in the predicted interactions and those related to bacterial stem blight. The eight *P. syringae* annotation modules can be accessed using the *Pseudomonas* dataset, while the results for the interactions module can be displayed by specifying the *Medicago* dataset.

**Figure 3:**
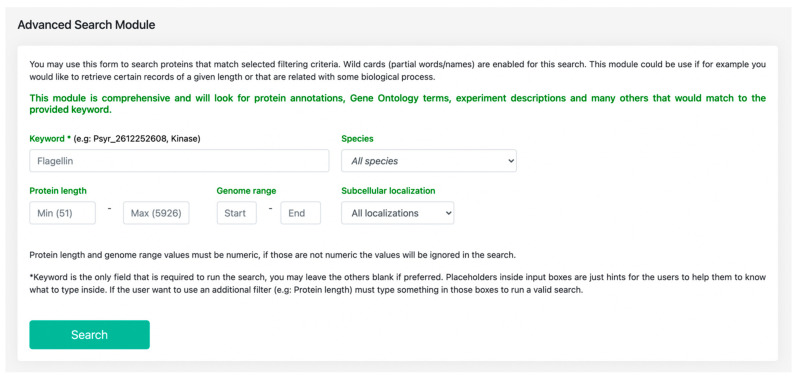
Advanced search module of alfaNET displaying the default parameters.

The database aims to provide comprehensive information on the *Pseudomonas* proteins to users through the ‘*advanced search*’ functionality (Figure 3). The interface of this module permits the user to specify the filtering parameters by using the keywords for the required species. As an extensive module, it incorporates the functioning of other modules, and thus searches for the given keyword in the respective backend tables to pull the particular record that matches the required criteria. Various information modules such as protein annotations, subcellular localization, and other annotations are queried in order to match with the specific keyword in question. Furthermore, the user can also obtain the protein accessions using the “basic” search option, which is easily accessible through all the modules of alfaNET.

**Figure 4:**
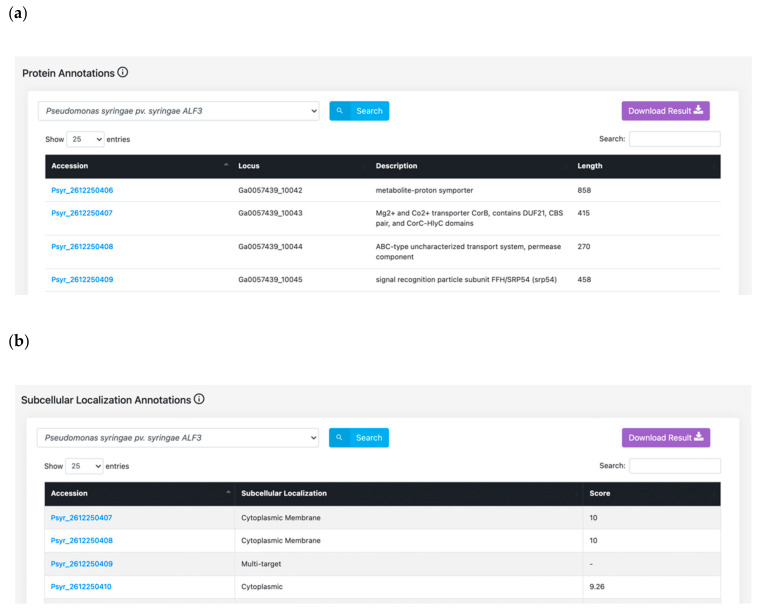

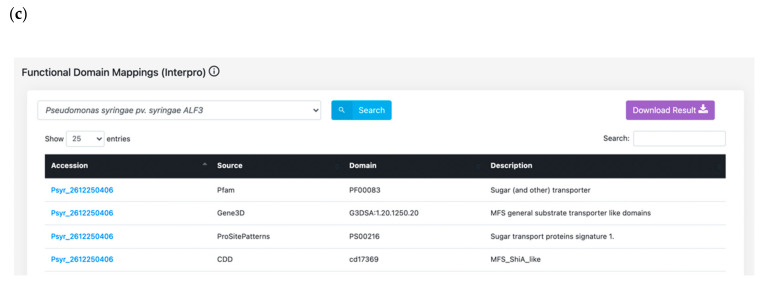
Examples of annotation modules of alfaNET: (**a**) protein annotations; (**b**) subcellular localizations; (**c**) functional domain mappings.

alfaNET is structured in such a manner that it can perform several tasks, for example: obtaining protein annotations of *P. syringae* proteins (Figure 4a); obtaining subcellular localization of the proteins (Figure 4b); and fetching functional domain mappings (InterPro) (Figure 4c) of the pathogen proteins related to bacterial stem blight. With the availability of these interfaces, we believe that this database will be a great resource for the research community.

### 3.2. Host-Pathogen Interactome: Towards a Better Understanding of Bacterial Stem Blight Infection Mechanisms

The study of PPIs provides an in-depth understanding of the biological functions of proteins [21]. Currently, the host–pathogen PPI prediction is primarily carried out using various experimental techniques such as yeast two-hybrid [22] and co-immunoprecipitation [23], among others. These techniques are primarily useful for the validation of few PPI pairs, but for large-scale PPI prediction such methods are expensive, time-consuming, and labor-intensive [24]. The advancements in high-throughput computational approaches have made the extensive prediction of PPIs rapid and tremendously efficient [25].

Accounting for the variations in bacterial strains, the PPI prediction for a particular pathogenic strain can contribute to the development of enhanced strain-specific treatment. Therefore, alfaNET implements an interactomics module to identify the PPIs between *P. syringae* and alfalfa that are responsible for causing bacterial blight disease. The interactomics tool allows the user to select protein–protein interaction databases, which serve as a reference for PPI prediction. The user can also define the filters for BLASTp alignments to determine the protein homologs. Furthermore, our database implements an efficient plugin, SigmaJS, for network visualization. This plugin is known for its enhanced performance and ability to illustrate large networks. The network visualization platform provides the user with detailed information about each node, such as species, degree, and description, along with the identification of hub nodes. The hub nodes are of high research interest, as these play a significant role in deciphering disease-related pathways and cellular processes [26]. Besides analyzing the network with this tool, the user can also download the resultant network files in JSON or SVG format, enabling the user to examine the networks in third-party network analyzer tools.

### 3.3. BLAST Server and Bulk Data Download

**Figure 5:**
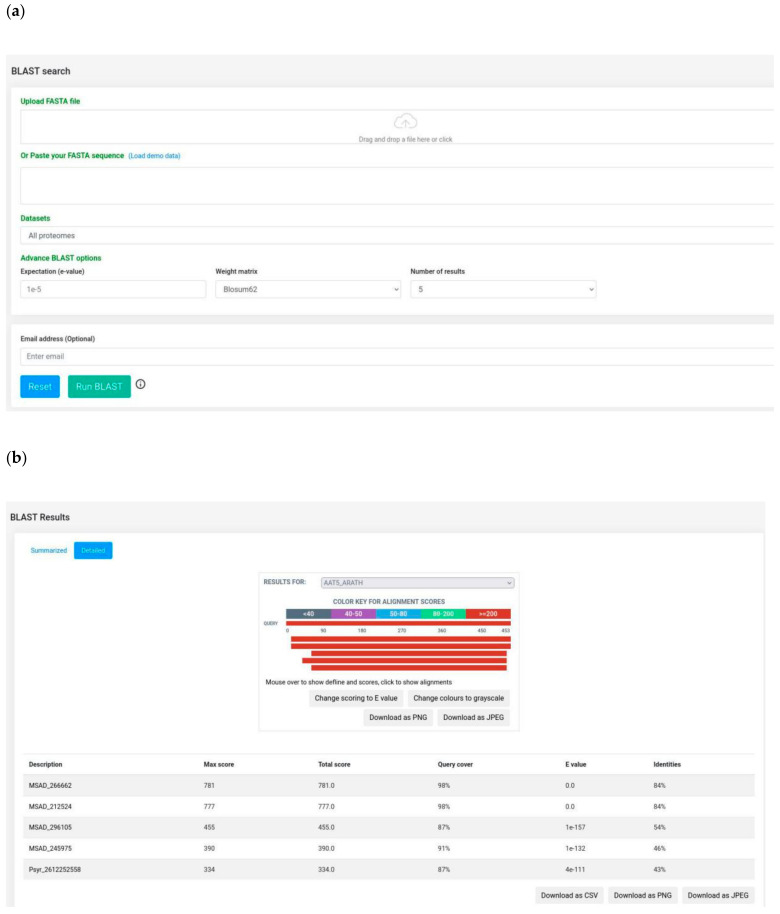
BLAST search module: (**a)** BLAST search interface; (**b**) visualization of BLAST alignments using BlasterJS.

The BLAST search module (Figure 5a) provides homology sequence search functionality to users. In this module, we implemented the NCBI’s BLAST locally on the server with the availability of two proteomes: alfalfa and *P. syringae*. The link to the protein sequences of the host and pathogen species used in alfaNET is available on the “*Datasets*” page, which forwards the user to the source of the dataset. The user has the choice to query sequences against the individual proteome or both (by default) the proteomes together at the same time. The module is of great advantage to users, as either the nucleotide or amino acid sequences can be uploaded as a query and the specific BLAST program (BLASTx or BLASTp) that needs to be performed is automatically detected by the system.

The “BLAST Results” page summarizes the information of BLAST alignments, which can be downloaded in various formats such as Excel or PDF, or can be copied to the clipboard. Additionally, the alignments can be visualized in an enhanced mode using the “Detailed” option (Figure 5b). This option allows the user to download the alignments in PNG or JPEG format.

### 3.4. Applicability of alfaNET: A Case Study on Resistant and Susceptible Plant Responses in Medicago sativa to Bacterial Stem Blight

**Figure 6:**
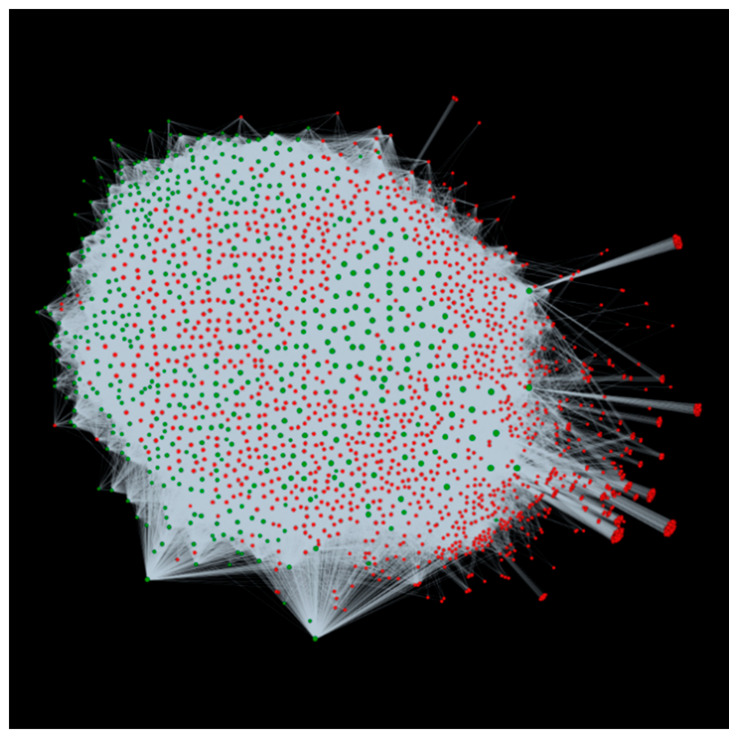
Visualization of the interactions for up-regulated genes of alfalfa interacting with *P. syringae*. Green nodes are host proteins and red nodes are pathogen proteins. Edges in grey represent the interactions from the interolog-based method.

**Figure 7:**
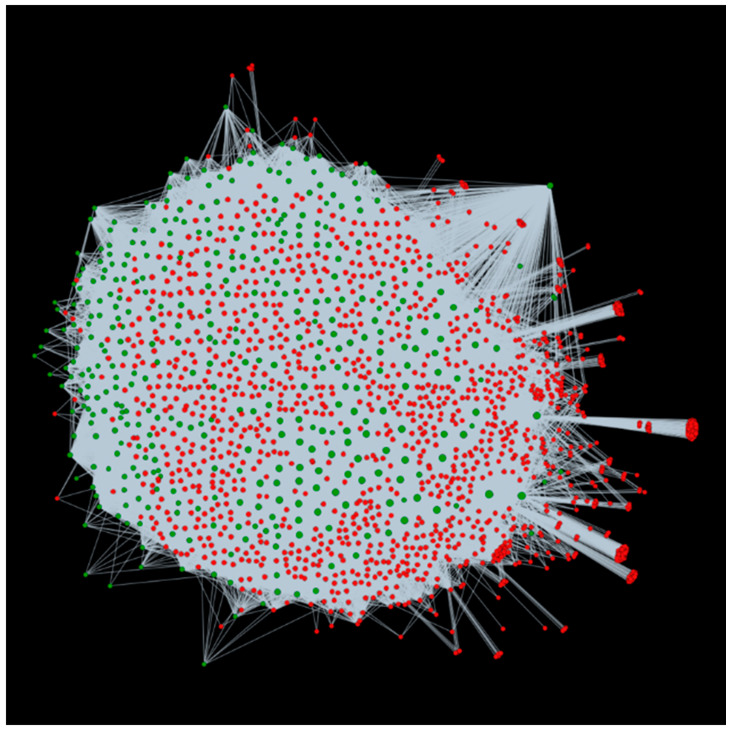
Visualization of the interactions for down-regulated genes of alfalfa interacting with *P. syringae*. Green nodes are host proteins and red nodes are pathogen proteins. Edges in grey represent the interactions from the interolog-based method.

In order to confirm the viability of alfaNET, we obtained the differentially expressed genes (DEGs) identified in resistant and susceptible plants of the alfalfa cultivar “Maverick”, infected with *P. syringae* pv. *syringae* ALF3, causing bacterial stem blight [12]. The bioinformatic analysis in this study revealed 810 genes up-regulated in resistant plants and 577 genes down-regulated in susceptible plants 72 hours after inoculation. We identified these DEGs in our PPIs obtained using the interolog-based computational approach and found 388 down-regulated genes and 542 up-regulated genes. The identified up-regulated genes were found to encode for plant resistance (R) genes, suggesting the defense response of the resistant plants. On the other hand, the down-regulated genes were involved in various functional categories such as protein phosphorylation, response to biotic stimulus, and transmembrane transport, thus being the reason for the plant becoming susceptible. Additionally, we also identified the interacting pathogens for these differentially expressed genes. A total of 1734 *P. syringae* proteins were found interacting with 542 up-regulated alfalfa genes (67% correctly identified), involved in 173,587 interactions (Figure 6). Meanwhile, for 388 down-regulated genes (67.24% correctly identified), 1457 *P. syringae* proteins were involved in 105,897 interactions (Figure 7). The annotations of the pathogen proteins are available in different modules of alfaNET. This shows the enrichment of alfaNET, and the identified genes can serve as a valuable dataset for biologists for further validation and characterization against bacterial stem blight disease.

To validate the interactions predicted using the case study above, the host–pathogen interaction pair “MSAD_210711-Psyr_2612254330” was further investigated. The functional analysis revealed that the host protein “MSAD_210711” is involved in the lipid catabolic process (GO:0016042), phospholipid metabolic process (GO:0006644), and glycerolipid metabolic process (GO:0046486). The enzymes involved in lipid biosynthesis are reported to play an essential role in various biotic and abiotic defense pathways. Phosphatidic acid (PA), a major component of lipid biosynthesis, is involved in abscisic acid-mediated stomatal closure during pathogen attack [27]. On the other hand, the pathogen protein “Psyr_2612254330” was found to be involved in the plant–pathogen interaction pathway (psb04626). The subcellular localization analysis showed that both the host and pathogen proteins are located in the cytoplasm, thus suggesting the site of protein–protein interaction. The above pair could be a good candidate for future experimental validations, among others.

### 3.5. Limitations and Future Development

The database implements multiple modules, a few of which consist of inadequate information about *P. syringae* pv. *syringae* ALF3 proteins due to the current availability of only the draft genome assembly, leading to incomplete annotation information. Predominantly, the polished genomes are used to perform molecular mechanism-related studies. Furthermore, to our knowledge, there is no previous report on the PPI prediction in the alfalfa-*P. syringae* system. In the future, we would probably enhance alfaNET by incorporating other *Pseudomonas* strains infecting alfalfa and include more experimentally validated data. Future developments will also include the addition of domain–domain interaction databases.

## 4. Materials and Methods

### 4.1. Data Source and Processing

The proteomes of alfalfa and *Pseudomonas syringae* pv. *syringae* ALF3 were retrieved from various sources. For alfalfa, the proteome was obtained from LegumeIP (https://plantgrn.noble.org/LegumeIP/gdp/, accessed on 10 November 2020) [28]. In total, 87,892 protein sequences were downloaded, which were further analyzed with CD-HIT [29] at 100% identity to reduce redundancy, leading to 87,156 proteins. The proteome of *Pseudomonas syringae* pv. *syringae* ALF3 was downloaded from JGI Integrated Microbial Genomes and Microbiomes (IMG/M) (https://img.jgi.doe.gov/, accessed on 10 November 2020; Taxon ID: 2609460275) and UniProt proteomes (https://www.uniprot.org/proteomes/, accessed on 30 July 2021; UP000028706). The *Pseudomonas* protein sequences from both the sources were combined and 100% identical sequences were clustered, leading to 5527 unique protein sequences. Since the cytoplasmic proteins of pathogens are believed not to take part in host–pathogen interactions, such proteins were eliminated [30]. Subsequently, the cytoplasmic proteins were analyzed at EffectiveDB (https://effectors.csb.univie.ac.at/, accessed on 16 December 2020) to predict the secreted proteins, and those predicted as secreted were included in the analysis. This approach gave 4427 *P. syringae* proteins for further analysis.

To employ the homology-based interolog approach, various PPI databases of The International Molecular Exchange Consortium (IMEX) [31] were used as templates. alfaNET implements five major databases from IMEX, *viz*., DIP 2020 [32], IntAct v4.2.16 [33], MINT 2018 [34], BioGRID v4.2.191 [35], and HPIDB v3.0 [36]. We also implemented all the plant species from the STRING v11.0 database [37]. In addition, the ArabHPI database was also included in alfaNET. The above-mentioned databases were downloaded from the respective sources and implemented as an individual MySQL table locally.

### 4.2. Host-Pathogen Interactome Comparison Tool

A novel module, known as an “interactomics” tool, was developed to compare PPIs between host and pathogen. This module implements the homology-based interolog approach at the backend. The interolog approach relies on the concept of transferring the conserved interactions between the species based on the sequence homology [38]. For example, if two proteins A and B interacting in an organism contain their orthologs in A′ and B′ in other organisms, then A-B and A′-B′ are considered to be an interolog [39]. Furthermore, the ortholog proteins obtained from the above-mentioned approach are queried against the PPI databases. If the host and pathogen proteins match with the corresponding interactions in the PPI databases, then the particular protein pair is anticipated to be interacting.

In alfaNET, we employed this method at the backend to predict the possible host–pathogen and protein–protein interactions between alfalfa and *P. syringae* proteins. In actuality, we aligned the proteomes of alfalfa and *Pseudomonas* individually against the six PPI databases using the BLASTp v2.7.1 tool with default parameters, thus obtaining six alignment files for each of the proteomes. Following the alignments, the interolog prediction is performed using our in-house R and python scripts, which call on various SQL functions to extract the data from the database tables. All these steps are executed after the submission of the job by a user. The process of ortholog match querying was accelerated by indexing the columns of the SQL tables for the PPI databases as interactor_A and interactor_B.

### 4.3. Protein Annotation

For *Pseudomonas* proteins, the detailed description was obtained from the *P. syringae* pv. *syringae* ALF3 annotations available at JGI IMG/M. The functional annotation, including the prediction of conserved domains and important sites, was carried out by analyzing the *P. syringae* proteome in InterProScan [40] using the “iprlookup” and “goterms” parameters. The subcellular localization of the *Pseudomonas* proteins was obtained using PSORTb [41]. Furthermore, the effectors for bacterial proteins were predicted using EffectiveDB, a database for retrieving bacterial secreted proteins [42].

### 4.4. Dataset Collection for P. syringae Effectors

The plant pathogenic bacteria subvert the host cell machinery and suppress the immune responses by deploying effector proteins into the plant cell using the type III secretion system (T3SS) [43]. To enhance the pathogen protein annotations, we extracted the known, potential, and virulence proteins for *P. syringae* from three different sources mentioned below. In total, 655 unique effectors (known T3SS effectors, virulence effectors, and predicted effectors) were procured and implemented in the database.

#### 4.4.1. Known T3SS Effectors

There are 50 known T3SS effector proteins reported in the literature, which were obtained from *Pseudomonas syringae* Genome Resources (http://www.pseudomonas-syringae.org/, accessed on 18 December 2020).

#### 4.4.2. Virulence Effectors

We obtained 8 virulence factors for *P. syringae* by searching its orthologs against *Pseudomonas aeruginosa* PAO1 (reference) from Pseudomonas genome DB (https://www.pseudomonas.com/, accessed on 5 January 2021).

#### 4.4.3. Predicted Effectors

To determine the type III secreted proteins of *P. syringae*, we extracted the pre-calculated potential effector proteins from EffectiveDB (https://effectors.csb.univie.ac.at/, accessed on 5 January 2021), using *P. syringae* pv. *syringae* B728a as a reference. A total of 641 proteins were obtained, which were classified as secreted proteins on the basis of their signal peptide, secretion chaperon binding site, or eukaryotic-like domains.

### 4.5. Dataset Collection for Ice-Nucleation Proteins

As mentioned earlier, INPs serve as initiators of bacterial stem blight. In line with this, we obtained the orthologs of the gene *Psyr1608*, which belongs to *P. syringae* pv. *syringae* B728a [44]. This gene is localized on the outer membrane of the bacteria and encompasses the ice-nucleation proteins octamer repeat. A total of 30 orthologs *Psyr1608* were procured from the Pseudomonas genome DB (https://pseudomonas.com/, accessed on 24 February 2021).

## 5. Conclusions

We developed alfaNET, an extensive framework that provides various annotation functionalities for *P. syringae* proteins. The database provides a platform namely, ‘*interactomics*’ tool—which predicts the protein–protein interactions of the alfalfa-*P. syringae* system by implementing an interolog-based computational approach. This tool also presents an enhanced network visualization, thus providing in-depth information on host–pathogen interactions. We believe that alfaNET will be a useful resource for the research community as well as plant pathologists/breeders, and that it will provide experimental biologists with a better understanding of the infection mechanisms of the disease.

## Data Availability

Not applicable.
